# A mutation in the centriole-associated protein centrin causes genomic instability via increased chromosome loss in *Chlamydomonas reinhardtii*

**DOI:** 10.1186/1741-7007-3-15

**Published:** 2005-05-31

**Authors:** Ivan Zamora, Wallace F Marshall

**Affiliations:** 1 Dept. of Biochemistry & Biophysics, University of California, San Francisco, 600 16th St., San Francisco, California, 9414, USA

## Abstract

**Background:**

The role of centrioles in mitotic spindle function remains unclear. One approach to investigate mitotic centriole function is to ask whether mutation of centriole-associated proteins can cause genomic instability.

**Results:**

We addressed the role of the centriole-associated EF-hand protein centrin in genomic stability using a *Chlamydomonas reinhardtii *centrin mutant that forms acentriolar bipolar spindles and lacks the centrin-based rhizoplast structures that join centrioles to the nucleus. Using a genetic assay for loss of heterozygosity, we found that this centrin mutant showed increased genomic instability compared to wild-type cells, and we determined that the increase in genomic instability was due to a 100-fold increase in chromosome loss rates compared to wild type. Live cell imaging reveals an increased rate in cell death during G1 in haploid cells that is consistent with an elevated rate of chromosome loss, and analysis of cell death versus centriole copy number argues against a role for multipolar spindles in this process.

**Conclusion:**

The increased chromosome loss rates observed in a centrin mutant that forms acentriolar spindles suggests a role for centrin protein, and possibly centrioles, in mitotic fidelity.

## Background

Centrioles are cylindrical structures located within the core of the centrosome. Although the localization of centrioles within the centrosome together with their precise duplication prior to mitosis has suggested a role in bipolar spindle assembly or function, the actual role of centrioles in cell division remains unclear and controversial. Cells from which centrioles and centrosomes are ablated can still form bipolar spindles via a centrosome-independent self-organization process, but the effectiveness of such spindles in terms of chromosome segregation has not been carefully measured. Based on the circumstantial evidence that tumor cells which display genomic instability also frequently show aberrations in centriole structure or copy number [1,2], it has been proposed that centrioles may participate in the maintenance of genomic stability. If centrioles play a role in chromosome segregation, then mutations in genes encoding centriolar proteins would be expected to result in genomic instability.

The EF-hand protein centrin [3] is one of the few identified centriolar proteins. Centrin is found in all eukaryotes, and is localized to centrioles in vertebrate cells [4], basal bodies in green algae [5], and spindle pole bodies (SPBs) in yeast [6,7]. Centrin is functionally required for centriole duplication [8,9] and inheritance [10]. RNAi experiments in mammalian and algal cells have indicated that reduction of centrin can result in cell division arrest [9] and cytokinesis failure [11]. However, it is unclear if these defects are indirectly caused by abnormal centriole structure or copy number, or if instead they represent pleiotropic effects due to the direct participation of centrin in the cell division process. Centrin also appears to be involved in many other cellular processes, including flagellar excision [12], nuclear mRNA export [13], cell membrane integrity [14] and homologous recombination [15]. Consistent with these multiple roles, centrin localization is not restricted to centrioles [4], and centrin has been identified as a component of nuclear pores and flagellar dynein complexes [16,17]. Centrin is thus a versatile molecule that has been adapted to perform multiple diverse cellular functions. Given the fact that centrin localizes to centrioles, which are potentially involved in genome maintenance, together with the fact that centrin is functionally involved in centriole duplication and segregation, an obvious question is whether centrin plays a role in genomic stability. In this report we address this question using a centrin mutation in the green alga *Chlamydomonas reinhardtii*.

## Results

### Genomic instability in the centrin mutant *vfl2-1*

We tested whether centrin plays a role in genomic instability using a genetic assay for loss of heterozygosity in *Chlamydomonas reinhardtii*. *Chlamydomonas *(*Chlamydomonas *refers to *Chlamydomonas reinhardti *throughout) is a unicellular green alga with genetics similar to yeast, but which, unlike yeast, has centrioles (known as basal bodies) that are virtually identical to centrioles of vertebrate cells. In *Chlamydomonas*, centrin polymerizes into long fibers that link the centrioles/basal bodies to the nucleus. These fibers are called the nucleus basal body connectors (NBBCs) or rhizoplasts [18,19]. The *vfl2-1 *mutant has a point mutation in the centrin gene [10], and these mutants produce a reduced quantity of centrin protein that is unable to polymerize into a rhizoplast. The *vfl2 *mutation appears to result in a defect in centriole segregation, because mutant cells have a variable number of centrioles (between zero and six per cell). Despite their centriole number variation, these mutants are viable and form seemingly normal bipolar mitotic spindles. It has been argued on this basis that centrin-based rhizoplasts do not play a role in mitosis [19]. Importantly, the centrioles in a *vfl2 *mutant cell are not located at the spindle poles [19], consistent with ultrastructural studies showing that, in green algae, centrioles are joined to the spindle poles by centrin-based fibers derived from the rhizoplast [20]. The high viability of *vfl2 *mutants thus allows us to measure genomic instability using genetic assays because populations of mutant cells can be grown for a sufficient number of generations to allow rare chromosome loss events to be detected.

**Figure 1:**
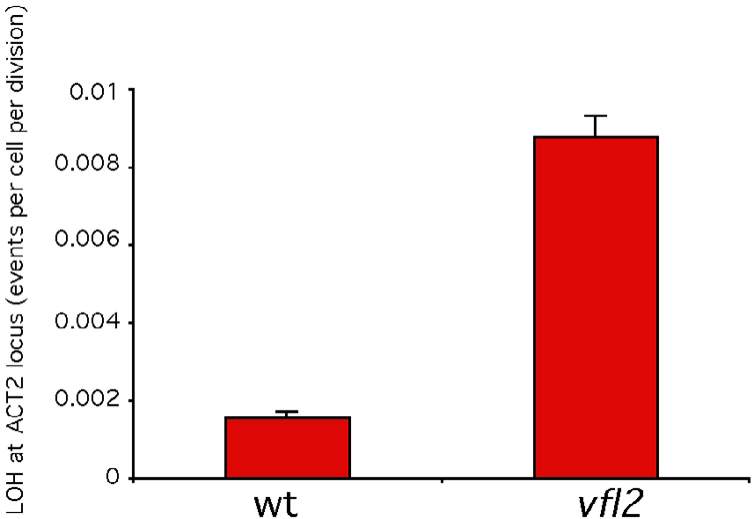
Genomic instability in the *Chlamydomonas *centrin mutant *vfl2*. Graph indicates frequency of loss of heterozygosity (LOH) at *ACT2 *test locus according to measurements based on the Luria-Delbrück fluctuation test. Error bars correspond to standard error of the mean.

We measured genomic instability in *vfl2 *mutants using a simple genetic assay for loss of heterozygosity (LOH) previously developed by Dutcher and coworkers [21]. This assay uses the *act2 *mutation, which confers recessive resistance to cycloheximide [22]. The *act2 *heterozygous diploids are sensitive to cycloheximide, while homozygous or hemizygous *act2 *diploids are cycloheximide-resistant. When a diploid culture is plated on media containing cycloheximide, only cells that have lost the wild-type *ACT2 *allele can grow. By measuring the frequency with which such cells arise, the rate of chromosome loss can be calculated (see Methods). As illustrated in Fig. 1, using 48 independently constructed diploid lines each for wild type and *vfl2*, we found that the LOH rate per cell division increased from 1.5 × 10^-3 ^± 1.6 × 10^-4 ^SEM (n = 48) in wild type, to 8.8 × 10^-3 ^± 5.7 × 10^-4 ^SEM (n = 48) in *vfl2 *mutants. This difference is statistically significant (*P *< 0.00005 based on one-tailed Welch's approximate *t*-test, *t *= 12.2). The increase in loss of heterozygosity in the *vfl2 *centrin mutant relative to wild-type cells indicates that centrin plays a role in genomic stability.

### Genomic instability occurs via chromosome loss

**Figure 2:**
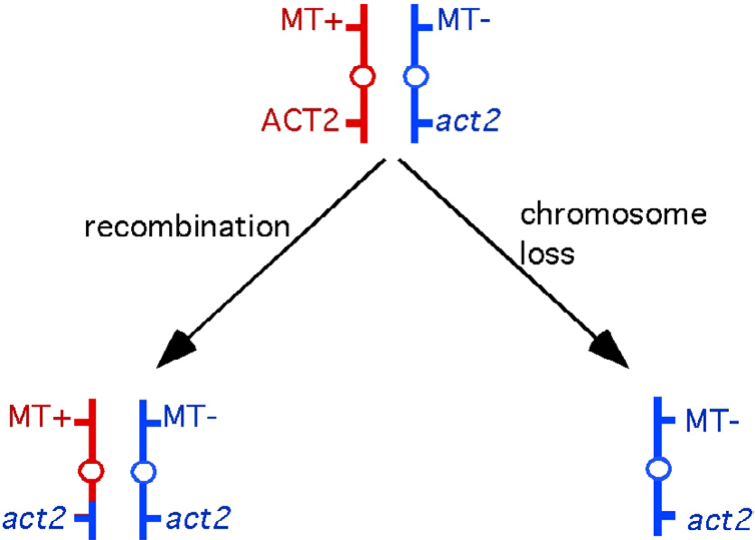
Pathways of *ACT2 *LOH. LOH due to mitotic recombination, including crossover and gene conversion events, will have no effect on the mating type locus *MT *on the other arm of the chromosome; hence cells will retain both the *MT+ *and *MT- *alleles. This is also true for spontaneous mutation of the *ACT2 *gene. In contrast, chromosome loss will result in simultaneous LOH at both *ACT2 *and *MT*.

In principle the LOH seen in *vfl2 *mutants could occur either by increased recombination or increased chromosome loss (Fig. 2). The latter would be consistent with a role for centrin or centrioles in mitotic spindle function, while an effect on recombination would suggest a spindle-independent role in DNA maintenance or chromosome dynamics. It is critical to distinguish these pathways because a role for centrin in DNA recombination and repair has been documented in plants [15]. Given the close phylogenetic relation between green algae like *Chlamydomonas *and green plants, the involvement of centrin in recombination in plants raises the real possibility that the loss of heterozygosity observed in *vfl2 *might have nothing to do with mitotic spindle function and might instead be driven by increased recombination.

In order to determine whether genomic instability in the *vfl2 *mutant is caused by increased recombination or chromosome loss, we distinguished between the two pathways by analyzing the mating type (*MT*) locus, which is located on the opposite arm of the same chromosome from *ACT2 *(Fig. 2). Diploid cells are heterozygous at *MT *and contain both the *MT+ *and *MT- *alleles. Because the *ACT2 *and *MT *loci are on opposite arms of the same chromosome, and recombination only causes LOH on a single arm distal to the crossover site, it can be inferred that only chromosome loss, and not mitotic recombination, could lead to the simultaneous loss of both the wild-type *ACT2 *gene and the *MT+ *allele (Fig. 2).

**Figure 3:**
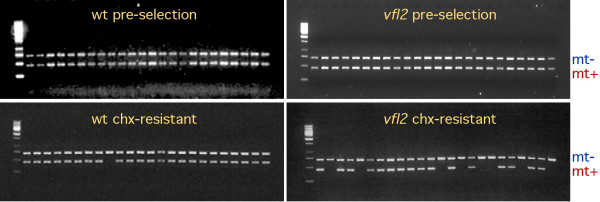
An increased proportion of LOH events is due to chromosome loss in *vfl2 *cells compared to wild-type cells. PCR analysis of *MT *locus before and after selection for LOH at *ACT2 *locus based on resistance to cycloheximide (chx) as described in Methods. Loss of *MT+ *specific band suggests that chromosome loss accompanied LOH at *ACT2*. Each panel shows 24 out of a total of 96 samples analyzed for each case.

We therefore tested lines that had undergone LOH at the *ACT2 *locus for chromosome loss versus recombination, by analyzing the *MT *locus using PCR primers specific for the *MT- *and *MT+ *alleles [23]. As illustrated in Fig. 3, prior to selection on cycloheximide, all wild-type and *vfl2/vfl2 *diploid lines were indeed diploid, as judged by the fact that they were heterozygous at the *MT *locus and contained both *MT+ *and *MT- *alleles as expected for a diploid. In wild type, of 96 independently constructed diploid lines selected on cycloheximide, only two lines showed concomitant loss of the *MT+ *allele and, therefore, chromosome loss. In contrast, of 96 cycloheximide resistant *vfl2 *lines, we found that 27 had also lost the *MT+ *allele, loss of which indicated that the lines had undergone chromosome loss. These chromosome loss events apparently represent the loss of individual chromosomes rather than an overall return to haploidy, as the cells were able to grow on media lacking arginine and thus seemed to have retained the two homologs of the chromosome containing the *arg2/arg7 *mutations used to select for diploidy (see Methods). The difference in the proportion of LOH events due to chromosome loss (2/96 for wild type vs. 27/96 for *vfl2*) was highly significant (*P *< 0.00001 based on Fisher's Exact Test). Multiplying the LOH rate by the fraction of LOH events due to chromosome loss, we found that the total rate of chromosome loss per cell division increased almost 100-fold, from 3.2 × 10^-5 ^in wild type to 2.4 × 10^-3 ^in *vfl2 *mutants.

### Cell death during G1 in *vfl2 *mutants

The fact that *vfl2 *mutants show increased chromosome loss in the genetic assay described above leads to the prediction that haploid *vfl2 *cells should frequently produce inviable progeny cells. This prediction follows from the fact that all *Chlamydomonas *chromosomes contain essential genes, so a single chromosome loss event for any of the 17 chromosomes should produce an inviable progeny cell when haploid cell division are analyzed. (Note: chromosome loss did not lead to death in the experiments described above because those assays were conducted using diploid cells. Thus, chromosome loss led only to monosomy rather than nullisomy.) Consistent with this prediction, analysis of cell viability using the vital stain phenosaffranin indicated that *vfl2 *cultures contain a higher fraction of inviable cells than wild type (data not shown).

**Figure 4:**
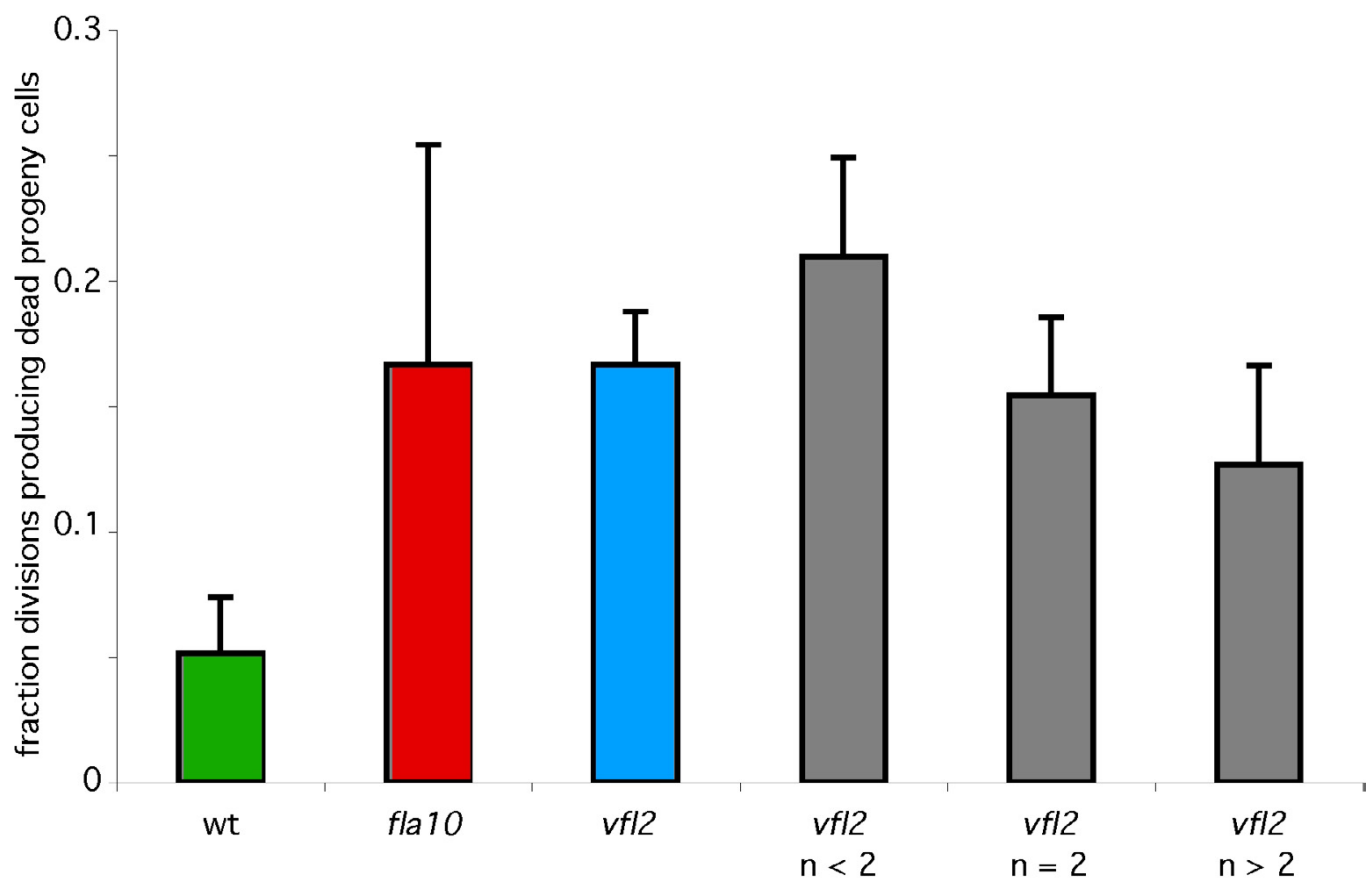
Production of inviable progeny cells during division in haploid *Chlamydomonas *cells. Graph shows fraction of cell divisions in which one or both progeny cells are inviable based on morphology. (Green) Wild type; n = 97. (Red) The *fla10 *mutant, previously shown to have elevated rate of chromosome loss; n = 18. Production of inviable progeny likely reflects spontaneous missegregation of chromosomes resulting in nullisomic progeny that die due to lack of essential genes. (Blue) The *vfl2 *mutant; n = 312. As predicted from chromosome loss measurements, this mutant shows an elevated rate of inviable progeny. (Grey) Outcomes for *vfl2 *mutant cells with less than two, two, or greater than two centrioles, as judged by flagellar number (numbers of cell divisions scored: less than two centrioles, n = 105; two centrioles, n = 136; greater than two centrioles, n = 71). Cells with supernumerary centrioles do not show an elevated rate of dead progeny production compared to cells with two or fewer centrioles.

In order to test this prediction more quantitatively, we examined the outcomes of individual cell division events. Synchronized cultures of haploid *Chlamydomonas *cells were embedded in agarose pads under a coverslip and imaged during G1 (as judged by time relative to the light-dark cycle and by the presence of flagella on most cells) by phase contrast microscopy as previously described [24]. Viable cells were identified and their position marked, and then their progeny were examined during the first G1 phase after division. Only cells that divided successfully to give two or four progeny were scored (note that sometimes, depending on cell size at the commitment point, *Chlamydomonas *cells can undergo two or three rapid division cycles during the dark phase of the circadian cycle). As a test of the methodology, we compared outcomes of division for wild-type cells and *fla10-1 *mutant cells grown at 21°C. The *fla10-1 *mutation occurs in a kinesin involved in flagellar assembly, and it has been reported that *fla10-1 *mutants show an increased rate of mitotic chromosome loss when grown at the permissive temperature [21]. If chromosome loss leads to increased production of inviable progeny, then this should be detectable in our analysis. Indeed, as shown in Fig. 4, *fla10-1 *cells showed a significant increase in the fraction of divisions producing inviable progeny.

We then applied this method to *vfl2 *mutant cells. As indicated in Fig. 4, we found that *vfl2 *showed a similar level of inviable progeny production as was observed in *fla10 *and significantly higher than in wild type. The cell death was observed during the first G1 after division and therefore did not result from a prolonged arrest or from a failure during subsequent S phase or mitosis. Dead cells were often roughly similar in size to their live siblings, suggesting that they were initially able to grow but eventually became inviable as they grew. In most cases the viable sibling cells retained flagella, indicating the culture had not yet reached the first S phase (at which time flagella are resorbed in *Chlamydomonas *preparatory to division), confirming that death occurred during G1. This cell death during G1 is consistent with increased chromosome loss during mitosis, which should result in death during growth as essential gene products become depleted. We cannot, however, rule out the possibility that the cell death results from other defects in cell division.

In order to test whether supernumerary centrioles might cause the elevated cell death we observed, we asked whether the cell division defects in *vfl2 *correlated with the number of centrioles present. As previously shown, the fact that all centrioles are active as basal bodies in *vfl2 *mutants allows the number of centrioles to be inferred from the number of flagella [24]. We therefore examined the outcome of cell divisions of cells having either the correct centriole copy number (n = 2), too few centrioles (n<2) or supernumerary centrioles (n>2). As indicated in Fig. 4, the fractions of inviable progeny were statistically similar for all three classes. We especially note that the cells with supernumerary centrioles did not show an increase in cell death after division.

## Discussion

These results demonstrate that the *vfl2-1 *point mutation [10] in centrin leads to increased genomic instability via an increase in chromosome loss, suggesting that centrin plays a role in mitotic chromosome segregation. Formally, these results suggest that the gene encoding centrin could act as a "housekeeper" type tumor suppressor gene, which when mutated would predispose a cell to tumorigenesis indirectly by increasing genomic instability.

The mechanism by which the *vfl2 *mutation leads to increased chromosome loss is unknown. The chromosome missegregation seen in the *vfl2 *centrin mutant is reminiscent of the Ipl (increase in ploidy) phenotype seen in yeast mutants defective in the yeast centrin homolog *CDC31 *[25], which is presumably due to a failure to form a mitotic spindle. By analogy with *cdc31 *mutants, it is then possible that the chromosome loss in *vfl2 *results from defective spindles. However, unlike in yeast where the spindle pole body is absolutely necessary to form a mitotic spindle, in higher eukaryotes it has been shown that bipolar spindles can self-assemble in the absence of centrioles or centrosomes [26]. Thus it was not obvious a priori that a defect in a centriole-associated protein (such as centrin) should have any effect on mitosis. Indeed, in the specific case of the *Chlamydomonas vfl2 *mutant, it has previously been reported that even though the *vfl2 *mutation causes centrioles to detach from the spindle poles, the acentriolar spindles are nonetheless still bipolar [19]. On this basis it was concluded by Wright and coworkers [19] that centrin does not play a role in mitosis. It might therefore seem surprising that we find an increase in chromosome loss in the *vfl2 *mutant. However, while the ability of cells lacking centrioles or centrosomes to form bipolar spindles is well documented, and these spindles can be seen to undergo an apparently normal anaphase chromosome separation [27], the question of whether such acentriolar spindles might have an altered fidelity of chromosome segregation compared to normal spindles has never been directly addressed. We speculate that the chromosome missegregation we observe in the *vfl2 *mutant reflects a decreased effectiveness of acentriolar spindles that self-assemble in this mutant. This would suggest a mitotic fidelity function for centrioles, which requires not merely the presence of centrioles in the cell, but also their specific localization at the spindle pole.

Alternatively, the chromosome loss we observe could be a byproduct of multipolar spindles. A small fraction (less than 1%) of *vfl2 *cells have three or more centrioles that could in theory give rise to tripolar or tetrapolar spindles. We suspect this is not the case because only bipolar spindles have been observed in these mutants [19]. Moreover, since centrioles are not located at spindle poles in this mutant, it is unlikely that the number of centrioles would have any effect on the number of spindle poles. The lack of centrioles at the poles also rules out configurations such as those seen during mitosis in multinucleate PtK1 cells [28], when multiple centriole pairs line up on either side of a single metaphase plate to form a masked multipolar spindle. Furthermore, to the extent that production of inviable cells during mitosis reflects chromosome loss, the data of Fig. 4 argue against multipolar spindles playing a dominant role in chromosome loss since cells containing supernumerary centrioles do not show any increase in the rate of production of inviable progeny cells.

It is also possible that the chromosome loss in *vfl2 *mutants arises through a spindle-independent mechanism. Because centrin may be involved in nuclear transport [13,17], it is possible that the *vfl2 *mutation could cause defects in chromosome organization due to a nuclear trafficking defect, leading indirectly to chromosome loss. We also note that disruption of centrin by RNAi in *Chlamydomonas *has been reported to cause cytokinesis defects leading to binucleate cells [11]. However such a cytokinesis defect cannot explain our results because the LOH assay we employed selects for loss of *ACT2*, rather than the presence of additional copies. Detailed cell-biological analysis of centrin mutant cells during division will be necessary to understand the basis of the chromosome loss we have observed.

## Conclusion

We have found that a mutation in the centriole-associated protein centrin, which causes affected cells to form acentriolar spindles and lack centrin-based fibers, produces an increase in genomic instability caused by an increase in the rate of chromosome loss. These results indicate that either centrioles or the centrin protein itself are involved in increasing the fidelity of mitotic chromosome segregation.

## Methods

Heterozygous *act2 *diploid cells were constructed by crossing haploid strains of genotypes *act2 arg7 mt- *× *ACT2 arg2 mt+*. The *arg2 *and *arg7 *mutations are present in the same gene but complement each other in trans, so that cells require both alleles in order to grow in media lacking arginine. The *ACT2 *and *ARG2/ARG7 *loci are on different chromosomes, allowing us to distinguish loss of an individual chromosome containing ACT2 from a general return to haploidy (based on the ability of cells to grow on media lacking arginine).

We inoculated *act2 *heterozygous diploid cultures in non-selective media at an average density of 1 cell per culture, and, after growth, plated them on media containing cycloheximide. We then calculated the rate of loss of heterozygosity (LOH) at *ACT2 *from the number of resistant cells and the number of cell divisions following the initial inoculation (determined from viable cell counts obtained by serial dilution) using the fluctuation test method of Luria and Delbrück [29].

## Authors' contributions

IZ conducted PCR-based assays for chromosome loss. WFM conceived and designed the experiments, constructed strains, conducted fluctuation tests to measure LOH rate, and conducted analyses of live cells during division. Both authors contributed to the writing of the manuscript, and both authors read and approved the final manuscript.
